# Conserved C-terminal tails link PilC1 and PilC2 to the *Kingella kingae* type IV pilus through β-strand augmentation with the core minor pilin complex

**DOI:** 10.1128/msphere.00464-26

**Published:** 2026-09-21

**Authors:** Taylor A. Yount, Eric A. Porsch, Alexa Mihaita, Aisling Macaraeg, Joseph W. St. Geme

**Affiliations:** 1 Perelman School of Medicine, University of Pennsylvania 6572, Philadelphia, Pennsylvania, USA; 2 Children’s Hospital of Philadelphia, Philadelphia, Pennsylvania, USA; The University of Arizona, Tucson, Arizona, USA

**Keywords:** *Kingella kingae*, type IV pili, PilC1, PilC2, adhesin, minor pilin, adherence, twitching motility, natural competence

## Abstract

*Kingella kingae* is a gram-negative bacterium that is a leading cause of septic arthritis, osteomyelitis, and a variety of other invasive diseases in young children. Infection begins with colonization of the oropharynx, which depends on type IV pili (T4P), surface appendages that promote adherence, twitching motility, and natural competence. These T4P functions are mediated by two pilus-associated proteins called PilC1 and PilC2, which are members of the PilC/PilY family of proteins. In this study, we used AlphaFold Multimer modeling to identify a conserved C-terminal tail in both PilC1 and PilC2 that is predicted to insert into a hydrophobic pocket in the FimT-PilV-PilW-PilX-PilE core minor pilin complex via β-strand augmentation. Truncation of the final 10 residues of PilC1 or PilC2 significantly impaired pilus biogenesis, adherence to epithelial cells, twitching motility, and natural transformation without altering PilC1 or PilC2 protein expression. Point mutations targeting conserved residues within the PilC2 C-terminal tail had little impact on pilus biogenesis but resulted in a marked reduction in adherence and natural transformation, as well as the elimination of twitching motility. AlphaFold modeling predicted that these conserved residues interact with a β-sheet in the globular domain of the minor pilin PilX. Consistent with this model, site-directed mutagenesis of the PilX β-strand residues predicted to contact PilC2 largely phenocopied the PilC2 C-terminal tail point mutations. Together, these findings establish that the C-terminal tail in PilC/PilY adhesins is a conserved adapter that links these adhesins to T4P through the core minor pilin complex, identifying a potential target for anti-adhesion therapeutic strategies.

**IMPORTANCE:**

Type IV pili (T4P) are dynamic, adhesive surface fibers that are produced by a diverse array of bacteria. A number of T4P systems contain a large pilus-associated protein in the so-called PilC/PilY family that promotes pilus biogenesis and mediates adhesive activity. The pediatric pathogen *Kingella kingae* produces PilC/PilY family proteins called PilC1 and PilC2, and expression of at least one of these proteins is necessary for the production of functional T4P. Using molecular modeling and mutagenesis approaches, we established that a short peptide at the C-terminal end of these proteins, which is conserved among PilC/PilY family members, is critical for PilC1 and PilC2 functions and mediates these functions via β-strand augmentation with a specific T4P-associated protein complex.

## INTRODUCTION

*Kingella kingae* is a gram-negative bacterium that causes invasive diseases primarily in young children between 6 months and 4 years of age (1). With the advent of molecular diagnostic techniques, *K. kingae* is now recognized as the most common cause of septic arthritis and osteomyelitis in this age group in many countries and is also an established cause of endocarditis in both children and adults (1). *K. kingae* is a common commensal of the oropharynx, with approximately 70% of children colonized at some point during the first 3 years of life (2). Invasive disease occurs following disruption of the oropharyngeal epithelium via a viral co-infection and/or the *K. kingae* pore-forming RtxA toxin, allowing bacteria to enter the bloodstream (3–5). Once in the bloodstream, *K. kingae* resists host immune defenses through expression of a polysaccharide capsule, a surface galactan homopolymer, and a factor H-binding protein (6–10).

*K. kingae* colonization of the oropharynx is believed to depend on type IV pili (T4P), dynamic, proteinaceous surface appendages that mediate adherence to epithelial cells, twitching motility, and natural competence (11, 12). T4P are major virulence factors in many human pathogens, including *Neisseria meningitidis*, *N. gonorrhoeae*, *Pseudomonas aeruginosa*, and *Legionella pneumophila*, among others (13–16). The T4P biogenesis machinery spans the gram-negative bacterial cell envelope and assembles the pilus through polymerization of small pilin subunits (~15 kDa) (12, 17, 18). In *K. kingae*, the PilA1 major pilin constitutes the bulk of the pilus fiber and functions primarily as a structural protein, as it lacks intrinsic adhesive activity (19). *K. kingae* T4P undergo extension and retraction, driven by the PilF and PilT cytoplasmic ATPases, which polymerize and depolymerize pilin subunits at the base of the fiber, respectively (11, 12).

The adhesive function of *K. kingae* T4P is mediated by two large proteins called PilC1 and PilC2, which belong to the PilC/PilY family of T4P-associated adhesins (11). These proteins directly promote pilus biogenesis, twitching motility, and natural competence and are essential virulence factors in multiple bacterial species (11, 13, 16). PilC/PilY proteins share a conserved C-terminal β-propeller domain and an N-terminal domain that harbors adhesive activity (11, 20–22). In *K. kingae*, expression of only the C-terminus of PilC1 or PilC2 is sufficient to promote surface piliation but not adherence or twitching motility, suggesting that the C-terminus has a role in anchoring these adhesins to the T4P fiber (11). However, the mechanism by which PilC1 and PilC2 are incorporated onto T4P remains unknown.

*K. kingae* produces five low-abundance pilin subunits, called minor pilins, that are encoded by the *fimTpilVpilWpilXpilE* operon, which is conserved across *K. kingae* isolates (19). Bacterial two-hybrid analyses combined with AlphaFold Multimer modeling revealed that FimT, PilV, PilW, PilX, and PilE may form a complex within T4P (19). Deletion of these core minor pilins results in a significant reduction in surface piliation and disruption of adherence, twitching motility, and natural transformation (19). In other T4P-expressing bacteria, homologous core minor pilin complexes localize to the pilus tip and serve as priming platforms for pilus assembly (23, 24). Cryo-electron microscopy analyses in *Myxococcus xanthus* suggest that the PilY adhesin co-localizes with the core minor pilins at the pilus tip, raising the possibility that this complex serves as a docking site for PilC/PilY family members (24). *K. kingae* is unusual in encoding two distinct PilC proteins with minimal sequence identity (7%) and similarity (16%) to one another, raising fundamental questions as to their respective roles, stoichiometry, and localization with respect to the pilus fiber (11, 12). Of note, the *pilA1*, *pilC1*, and *pilC2* genes and the *fimTpilVpilWpilXpilE* operon are present at unlinked locations in the *K. kingae* genome (19). The localization of PilC1 and PilC2 and the mechanism of loading of PilC1 and PilC2 onto T4P have not been defined in *K. kingae*.

In this work, we used a combination of *in silico* modeling, site-directed mutagenesis, and functional assays to identify a conserved C-terminal tail in both PilC1 and PilC2 that is required for surface piliation and T4P-dependent adherence, twitching motility, and natural transformation. In addition, we defined conserved residues within the PilC2 C-terminal tail that are predicted to bind the core minor pilin complex and showed that these residues are critical for PilC2 function, facilitating β-strand augmentation with the PilX minor pilin. Point mutations in the PilX β-strand that are predicted to interact with the PilC2 C-terminal tail also attenuated or eliminated T4P-dependent phenotypes, mimicking the strain expressing the PilC2 C-terminal tail point mutations. Notably, the *K. kingae* PilC1 and PilC2 C-terminal tails exhibit both sequence and structural similarity to homologs in other T4P-expressing bacteria, suggesting that these C-terminal tails may be critical for docking PilC/PilY family members to the pilus via the core minor pilin complex in diverse T4P-expressing bacteria.

## RESULTS

### AlphaFold-predicted C-terminal tails are conserved in PilC/PilY family members and are essential for proper pilus biogenesis in *K. kingae*

**Fig 1:**
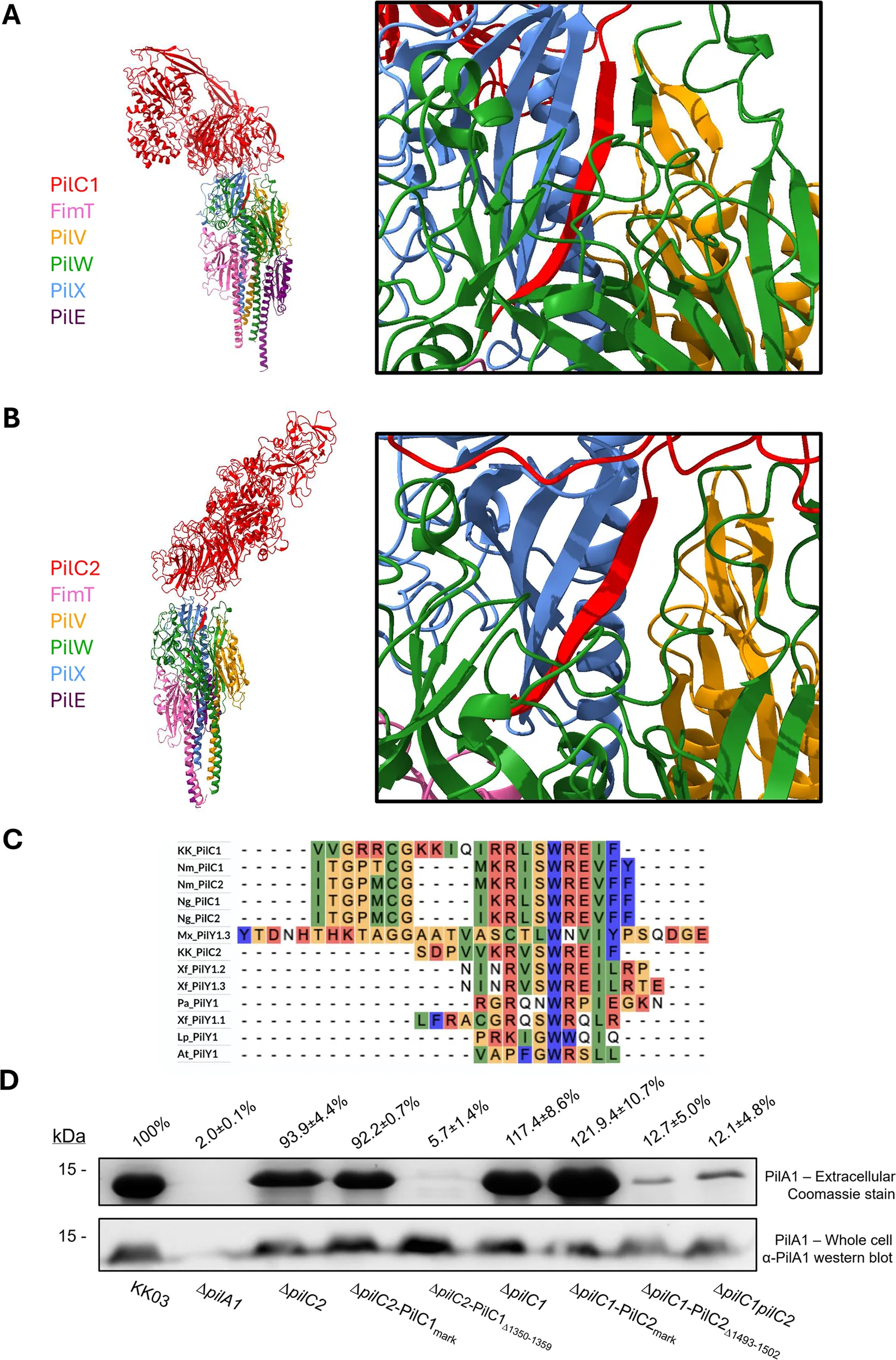
Predicted PilC1 and PilC2 C-terminal tails are conserved and are critical for T4P biogenesis. AlphaFold Multimer was used to predict the structures formed by the mature core minor pilins (FimTPilVWXE) and PilC1 (**A**) or PilC2 (**B**) in *K. kingae*. The legend on the left corresponds to the colors of each protein in the structure. On the right, there is a zoomed-in depiction of the C-terminal tail of either PilC1 (**A**) or PilC2 (**B**) to demonstrate the insertion of the tails into the core minor pilin complex and their interaction with specific minor pilins in the binding pocket. The AlphaFold confidence scores are listed in Table S1. (**C**) A Clustal Omega multiple sequence alignment was performed to align the terminal 50 amino acids for each of the PilC/PilY family proteins described in the literature. Homologs of the PilC/PilY family were taken from the reference strains for *M. xanthus* (Mx), *K. kingae* (Kk), *N. meningitidis* (Nm), *N. gonorrhoeae* (Ng), *Xylella fastidiosa* (Xf), *P. aeruginosa* (Pa), *L. pneumophila* (Lp), and *Acidithiobacillus thiooxidans* (At). The colors for the amino acid residues correspond to the properties of the amino acids and are color-coded using the standard ClustalW color scheme, where green represents nonpolar, hydrophobic residues, yellow represents small uncharged residues, red represents charged residues, and blue indicates aromatic residues. Dashed lines indicate gaps in the alignment. (**D**) Purified pili (top, Coomassie-stained gel) and whole-cell lysates (bottom, α-PilA1 western blot) from *K. kingae* strains were boiled and separated by SDS-PAGE to measure extracellular pili. The gel and blot analyses were performed three independent times, and representative images are shown. Densitometry was performed on each of the three replicate extracellular pilus preparation gels, and the band intensities were normalized to 100% for wild-type KK03. The densitometry quantification values are displayed as a percentage of KK03 ± standard error of the mean.

AlphaFold-predicted structures suggest that *K. kingae* PilC1 and PilC2 dock to the previously identified FimT-PilV-PilW-PilX-PilE minor pilin complex via their C-termini (19) (Fig. 1A and B). Closer examination of these structural models revealed that the final 10 amino acids in the C-termini of both PilC1 and PilC2 are predicted to extend into a pocket at the top of the minor pilin tip complex (Fig. 1A and B), raising the possibility that these C-terminal “tails” promote stable incorporation of PilC1 and PilC2 onto the pilus fiber. A multiple sequence alignment of the C-terminal ends of several PilC/PilY family members showed that there is a conserved tryptophan present in each PilY family member (Fig. 1C; Fig. S1). In many of these PilC/PilY proteins, the tryptophan residue is followed by a positively charged arginine, a negatively charged glutamate, and then a string of hydrophobic residues including isoleucine, valine, leucine, and phenylalanine (Fig. 1C; Fig. S1). Given the known variation in overall sequence similarity and identity among the PilC/PilY family, it is striking that the C-terminal tails display such high similarity to one another (21). To examine whether the sequence conservation of these C-terminal tails is associated with shared structural features, we generated AlphaFold Multimer models of PilC/PilY family members in complex with their corresponding core minor pilins (Fig. S2). Across *K. kingae*, *Neisseria* spp., *P. aeruginosa*, *M. xanthus*, and *L. pneumophila*, the models predicted that the PilC/PilY proteins extend a C-terminal tail into the top of a minor pilin complex, where the tail aligns closely with the β-sheet region of the PilX/PilK globular domain (Fig. S2). This arrangement is consistent with a β-strand augmentation interface, a type of protein-protein interaction previously described in other pilus systems and known to support high mechanical stability (25–27). Together, these observations led us to hypothesize that the C-terminal tails of PilC/PilY proteins mediate a structurally conserved and functionally important docking interaction at the pilus tip.

To test this hypothesis, we used site-directed mutagenesis to delete the final 10 residues of the C-termini of both PilC1 and PilC2 in *K. kingae* strain KK03, a stable derivative of septic arthritis clinical isolate 269-492 that expresses high levels of T4P (Fig. S3). All mutants derived from wild-type strain KK03 that are analyzed in this work harbor chromosomally-encoded mutations. AlphaFold-predicted models indicate that removal of the C-terminal tail is not expected to alter the overall fold of the PilC β-propeller domains, suggesting that this truncation is unlikely to disrupt the global structure of the proteins (Fig. S3). Importantly, there was no difference in PilC1 or PilC2 expression in cell lysates from the truncated mutants (Fig. S4). To assess the impact of the C-terminal tails on pilus biogenesis, surface pili were sheared and purified from each mutant. Consistent with previous observations (11, 28), strains KK03, Δ*pilC1*, and Δ*pilC2* displayed high levels of PilA1 within the purified pili samples (Fig. 1D). Strains Δ*pilC2*-PilC1_mark_ and Δ*pilC1*-PilC2_mark_, which contain an antibiotic resistance marker upstream of the wild-type *pilC1* gene or downstream of the wild-type *pilC2* gene, respectively, were used as positive controls when evaluating Δ*pilC2*-PilC1_Δ1350–1359_ and Δ*pilC1*-PilC2_Δ1493–1502_. These two control strains are necessary, as an antibiotic cassette marker is utilized for selection when introducing the desired mutations into *pilC1* and *pilC2* on the chromosome (see Materials and Methods). Both Δ*pilC2*-PilC1_mark_ and Δ*pilC1*-PilC2_mark_ displayed levels of PilA1 comparable to levels in KK03, Δ*pilC1*, and Δ*pilC2*, suggesting that insertion of the antibiotic resistance markers did not affect surface piliation (Fig. 1D). In contrast, Δ*pilC2*-PilC1_Δ1350–1359_ and Δ*pilC1*-PilC2_Δ1493–1502_ showed a significant decrease in PilA1 levels compared to their antibiotic resistance-marked controls, suggesting that the C-terminal tails of PilC1 and PilC2 promote surface piliation (Fig. 1D). Consistent with the prediction that the C-terminal tails are required for PilC1 and PilC2 loading onto the pilus, the piliation levels of Δ*pilC2*-PilC1_Δ1350–1359_ and Δ*pilC1*-PilC2_Δ1493–1502_ appeared comparable to the levels observed in a strain lacking both adhesins (Δ*pilC1pilC2*) (Fig. 1D). Importantly, there was no difference in PilA1 levels observed in cell lysates from these strains, suggesting that the observed decreases in PilA1 levels are likely due to defects in pilus biogenesis (Fig. 1D).

### *K. kingae* PilC1 and PilC2 C-terminal tails are essential for T4P-mediated adherence to host cells

**Fig 2:**
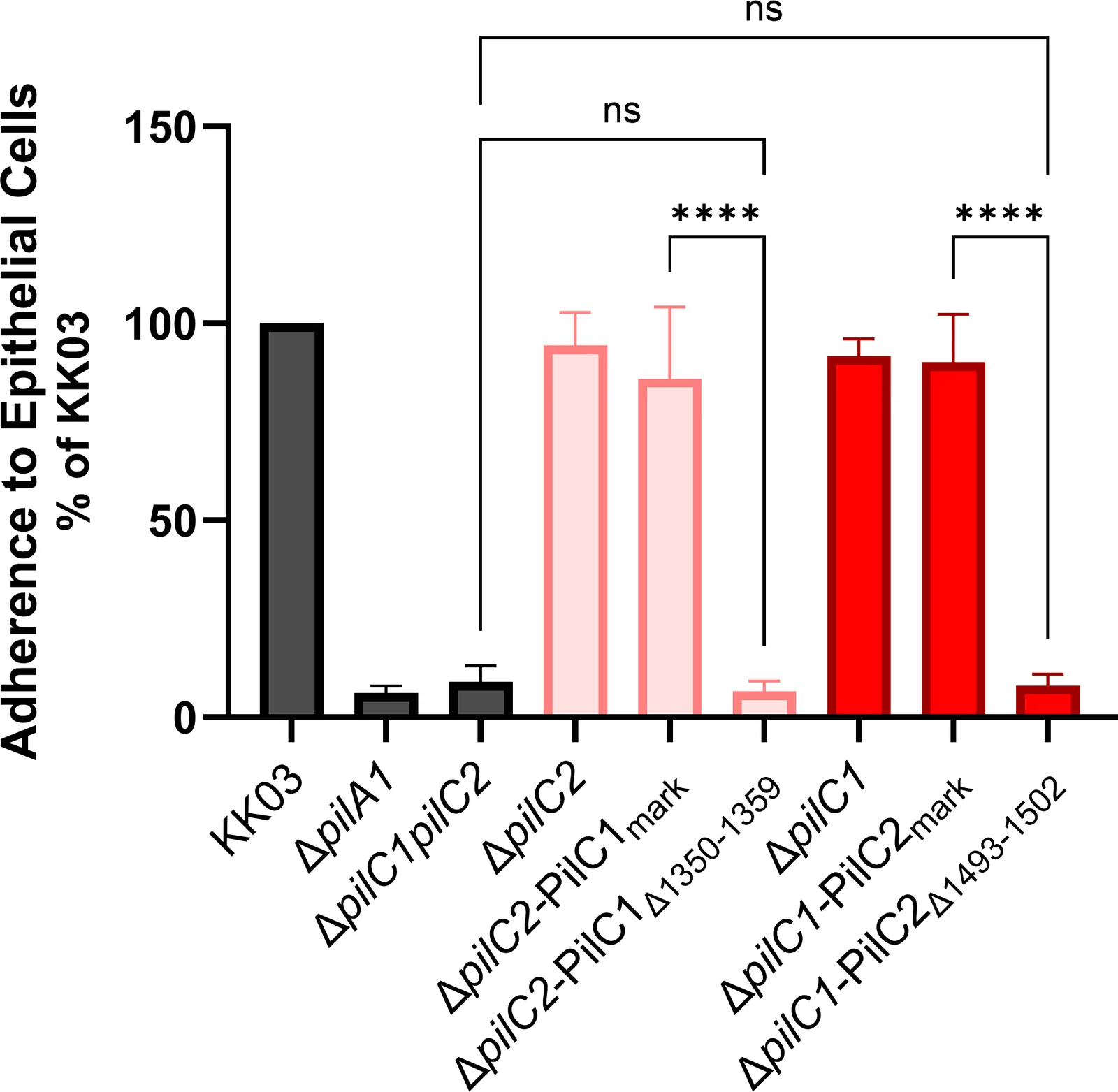
PilC1 and PilC2 C-terminal tails are critical for *K. kingae* adherence to host cells. Quantitative adherence assay with PilC1 and PilC2 C-terminal truncation mutants. *K. kingae* strains were added to monolayers of Chang epithelial cells of HeLa origin and evaluated for adherence using CFU plating. Adherence levels are reported as a percentage of wild type (KK03). The KK03 average adherence as a percentage of the inoculum = 41.8%. Error bars represent standard errors of the mean (*n* = 3 biological replicates). The symbol “****” indicates significance of *P* < 0.0001 as determined by one-way analysis of variance with a Bonferroni correction for multiple comparisons, and ns indicates no statistical difference.

*K. kingae* PilC1 and PilC2 directly mediate adherence to epithelial cells (11). To test whether the C-terminal tails of these adhesins are required for adherence, we performed adherence assays with Chang epithelial cells and the truncated PilC1 and PilC2 mutants. As previously described (11, 12, 28), strains that are non-piliated (Δ*pilA1*) or lack PilC1 and PilC2 (Δ*pilC1pilC2*) displayed negligible levels of adherence compared to wild-type KK03 (Fig. 2). There was no significant difference in adherence between strains that expressed just PilC1 (Δ*pilC2*) or just PilC2 (Δ*pilC1*) and the wild-type strain KK03 (Fig. 2). The antibiotic resistance-marked controls, Δ*pilC2*-PilC1_mark_ and Δ*pilC1*-PilC2_mark_, also demonstrated no differences in adherence compared to wild-type KK03 (Fig. 2). However, both Δ*pilC2*-PilC1_Δ1350–1359_ and Δ*pilC1*-PilC2_Δ1493–1502_ exhibited negligible levels of adherence, suggesting that the C-terminal tails of both PilC1 and PilC2 are required for T4P-mediated adherence to epithelial cells (Fig. 2).

### *K. kingae* PilC1 and PilC2 C-terminal tails are essential for T4P-mediated twitching motility

**Fig 3:**
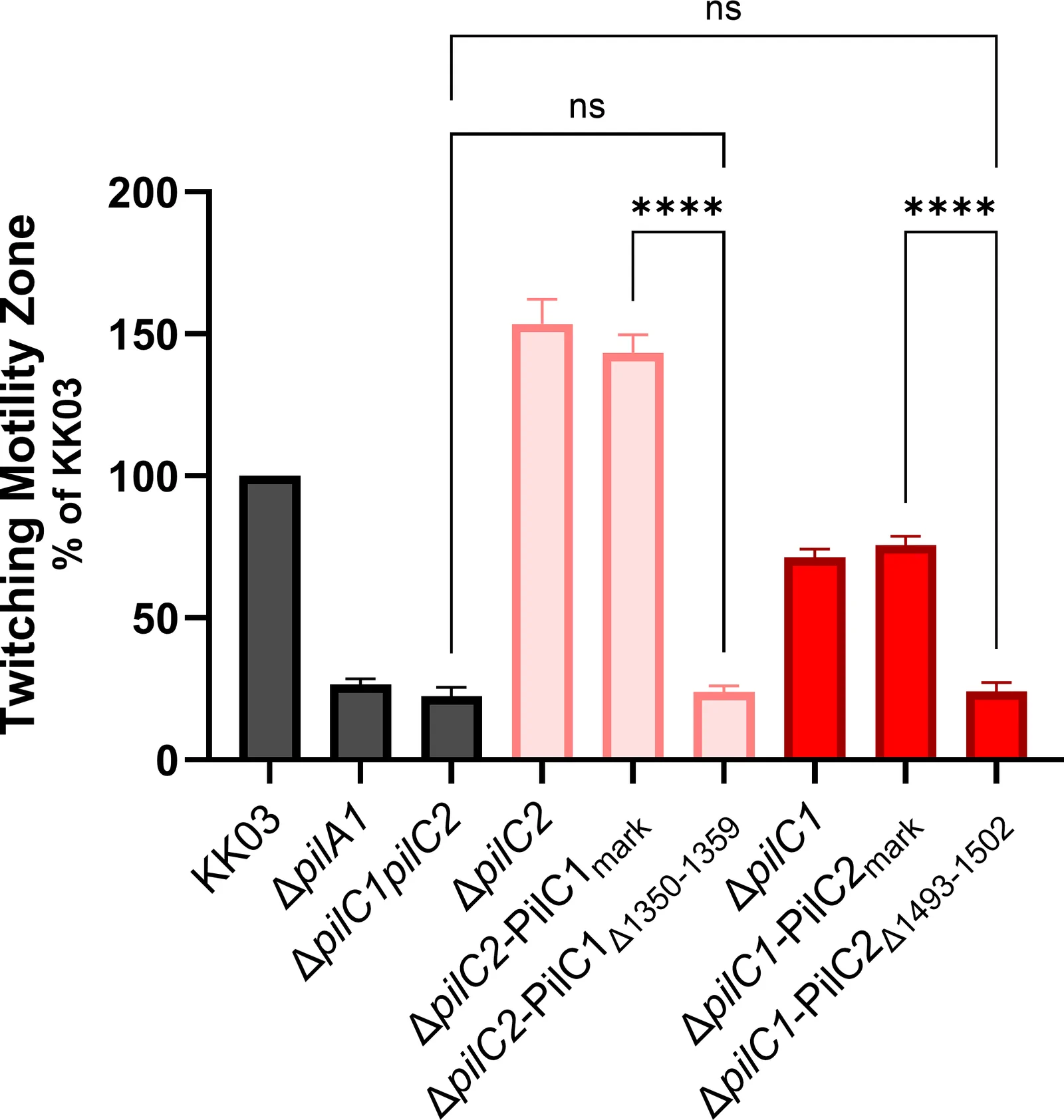
PilC1 and PilC2 C-terminal tails are critical for *K. kingae* twitching motility. Twitching motility assays with PilC1 and PilC2 C-terminal truncation mutants. *K. kingae* strains were stab inoculated into the center of low-agar chocolate plates, and the zone of twitching was quantified by measuring the diameter of bacterial spread as visualized by crystal violet staining. The average diameter of the twitching zones is reported as a percentage of wild type (KK03). The KK03 average twitching motility diameter = 25.1 mm. Error bars represent standard errors of the mean (*n* = 3 biological replicates). The symbol “****” indicates significance of *P* < 0.0001, as determined by one-way analysis of variance with a Bonferroni correction for multiple comparisons, and ns indicates no statistical difference.

PilC1 and PilC2 have also been shown to promote twitching motility, a form of surface motility driven by T4P. To evaluate the importance of the PilC C-terminal tails in twitching motility, *K. kingae* strains were stab-inoculated into the center of low-agar twitching plates, and the diameter of the zone of spread was measured after 72 h as a quantitative assessment of twitching motility. A non-piliated strain (Δ*pilA1*) and a strain that lacks both PilC1 and PilC2 (Δ*pilC1pilC2*) were used as controls and demonstrated no twitching on the agar plates (Fig. 3). Consistent with previous studies (11, 28), a strain expressing only PilC1 (Δ*pilC2*) exhibited a hypermotility phenotype with twitching levels 1.5-fold greater than wild type, and a strain that expresses only PilC2 (Δ*pilC1*) exhibited slightly lower levels of twitching motility compared to wild-type KK03 (Fig. 3). Both Δ*pilC2*-PilC1_Δ1350–1359_ and Δ*pilC1*-PilC2_Δ1493–1502_ displayed negligible levels of twitching motility that were comparable to the non-piliated control, suggesting that the C-terminal tails of both PilC1 and PilC2 are required for twitching motility (Fig. 3).

### *K. kingae* PilC1 and PilC2 C-terminal tails are essential for PilC-mediated natural competence

**Fig 4:**
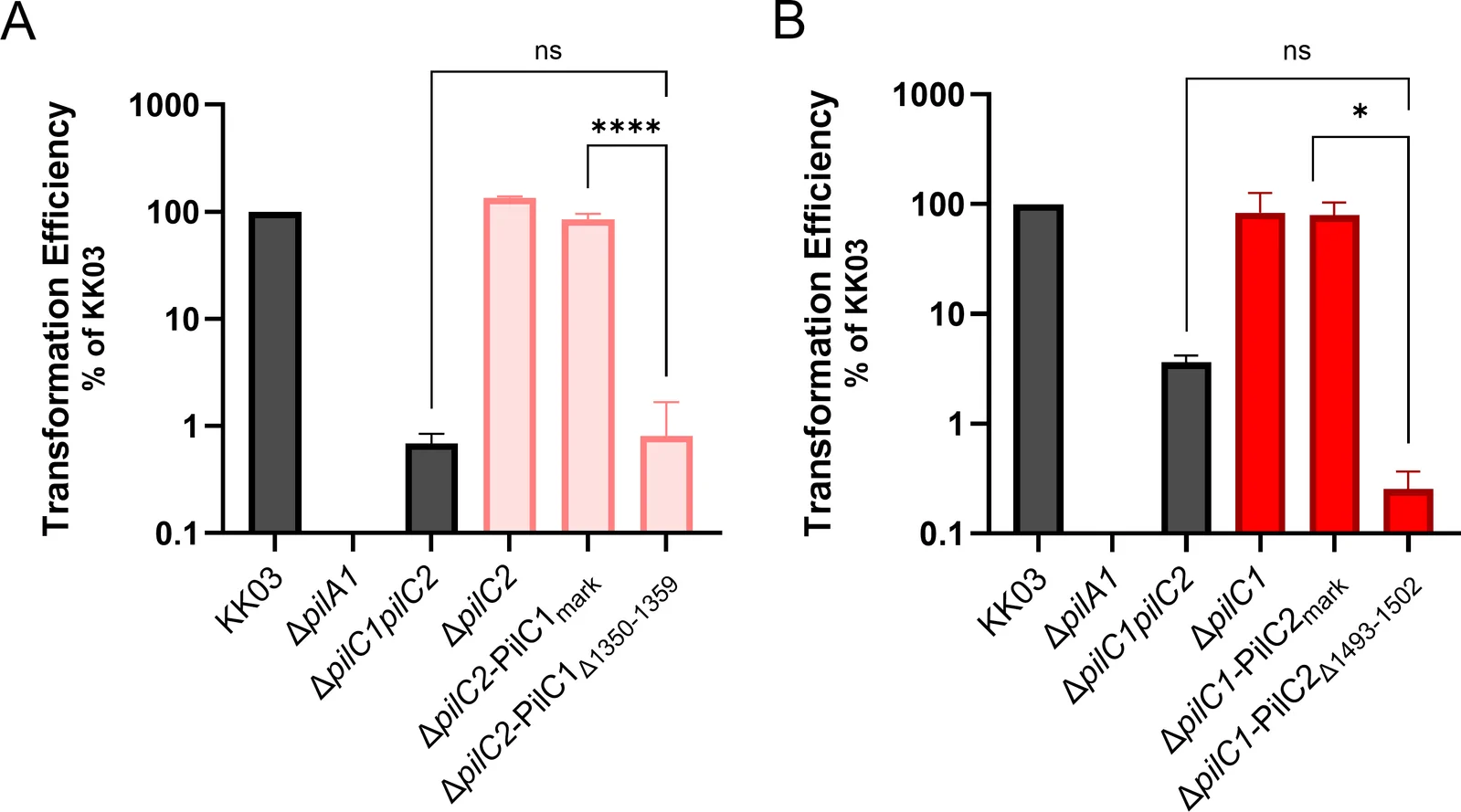
PilC1 and PilC2 C-terminal tails are essential for full-level *K. kingae* natural transformation. *K. kingae* strains were naturally transformed with a plasmid containing a kanamycin (**A**) or erythromycin (**B**) resistance cassette designed to replace the *knh* gene in the chromosome. Transformation efficiency was calculated as a ratio of antibiotic-resistant CFU to inoculum CFU. The transformation efficiency is reported as a percentage of wild type (KK03). For reference, the KK03 absolute transformation efficiency is 0.40% for panel **A** and 0.18% for panel **B**. Error bars represent standard errors of the mean (*n* = 3 biological replicates). The symbol “*” indicates significance of *P* < 0.05, and “****” indicates significance of *P* < 0.0001 as determined by one-way analysis of variance with a Bonferroni correction for multiple comparisons. ns indicates no statistical difference.

PilC1 and PilC2 are necessary to promote high levels of natural competence in *K. kingae* (11). To assess the contribution of the PilC C-terminal tails to natural competence, we performed natural transformation assays (Fig. 4). As expected, the non-piliated strain Δ*pilA1* was unable to take up exogenous DNA and incorporate an antibiotic resistance cassette into its genome (Fig. 4). Deletion of both PilC1 and PilC2 (Δ*pilC1pilC2*) resulted in a profound defect in natural transformation, with transformation efficiency reduced to <1% of wild-type KK03 levels (Fig. 4A). In contrast, strains expressing either PilC1 alone (Δ*pilC1*) or PilC2 alone (Δ*pilC2*) exhibited wild-type transformation efficiencies, consistent with known functional redundancy between PilC1 and PilC2 (Fig. 4A and B) (11). Strains expressing C-terminal truncation mutations of PilC1 (Δ*pilC2*-PilC1_Δ1350–1359_) or PilC2 (Δ*pilC1*-PilC2_Δ1493–1502_) showed marked reductions in transformation efficiency relative to the antibiotic resistance-marked controls (Fig. 4A and B). Transformation efficiencies in these truncation mutants were comparable to those observed in the Δ*pilC1pilC2* strain, suggesting that the C-terminal tails of PilC1 and PilC2 are essential for PilC-mediated natural transformation (Fig. 4A and B).

### Conserved amino acid residues in the PilC2 C-terminal tail and predicted interacting amino acids in the minor pilin PilX are critical for T4P biogenesis and function

**Fig 5:**
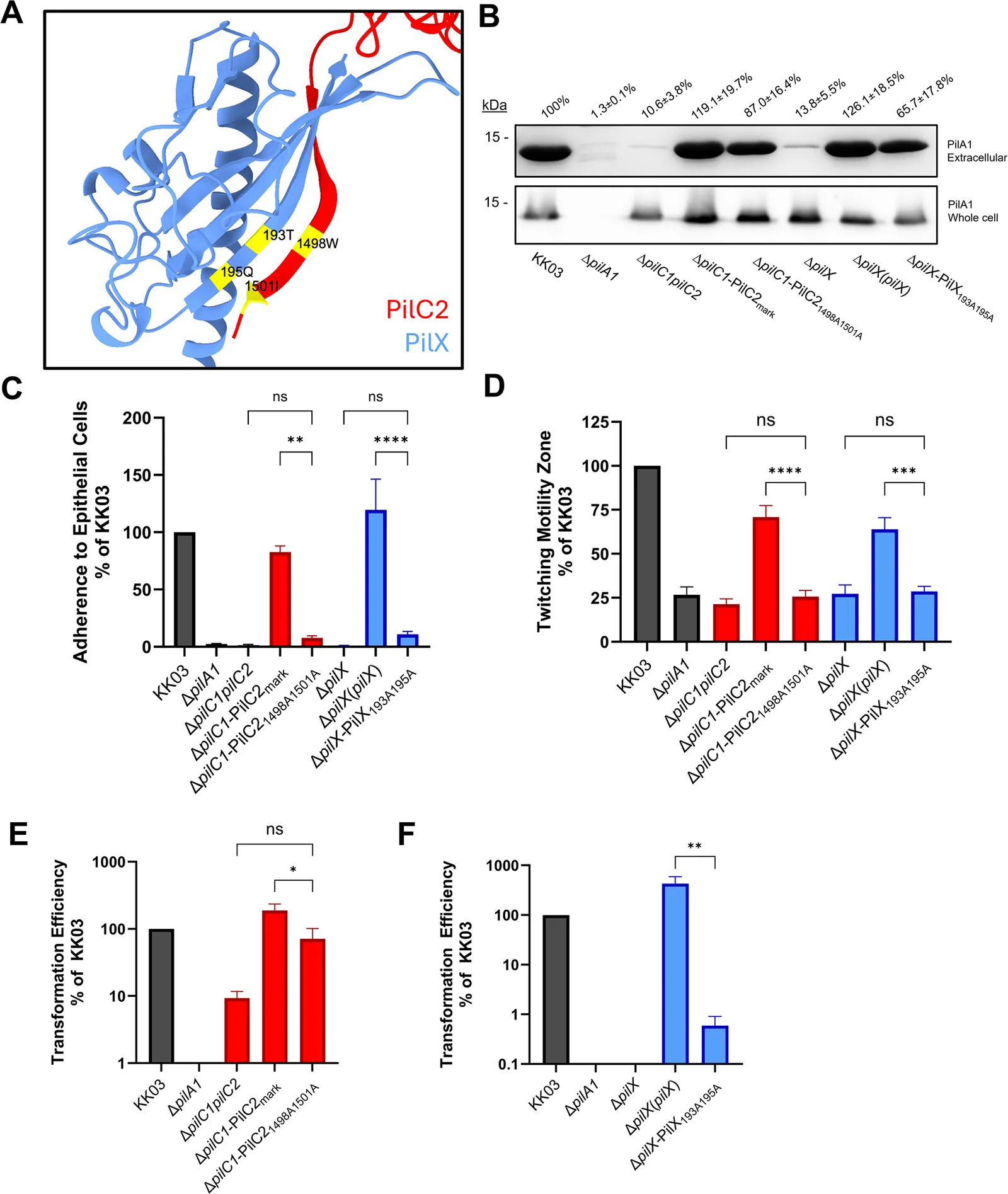
PilC2 and PilX point mutations impact T4P phenotypes. (**A**) The AlphaFold Multimer predicted β-strand augmentation of PilX (blue) and the PilC2 C-terminal tail (red), highlighting the residues targeted for mutagenesis (yellow), is shown. The AlphaFold confidence scores are listed in Table S1. (**B**) Paired pilus preparations (top, Coomassie-stained gel) and whole-cell lysates (bottom, α-PilA1 western blot) are shown. The gel and blot analyses were performed three independent times, and representative images are shown. Densitometry was performed on each of the three replicate extracellular pilus preparation gels, and the band intensities were normalized to 100% for wild-type KK03. The densitometry quantification values are displayed as a percentage of KK03 ± standard error of the mean. Adherence to epithelial cells (**C**) (KK03 average adherence as percentage of the inoculum = 77.4%), twitching motility (**D**) (KK03 average twitching motility diameter = 22.5 mm), and natural transformation (**E, F**) (KK03 average transformation efficiency = 7.71% for panel E and 1.38% for panel F) of strains expressing double point mutant PilC2 (∆*pilC1*-PilC2_1498A1501A_) and double point mutant PilX (∆*pilX*-PilX_193A195A_), along with the associated control strains, are shown. Error bars represent standard errors of the mean (*n* = 3 biological replicates). The symbol “*” indicates significance of *P* < 0.05, “**” indicates significance of *P* < 0.01, “***” indicates significance of *P* < 0.001, and “****” indicates significance of *P* < 0.0001 as determined by one-way analysis of variance with a Bonferroni correction for multiple comparisons. ns indicates no statistical difference.

As shown in Fig. 1C and Fig. S1, a multiple sequence alignment of PilC/PilY family members revealed a conserved motif within the *K. kingae* PilC1 and PilC2 C-terminal tails. Notably, for nearly every aligned protein, the C-terminal tail contained a conserved tryptophan residue followed by one or two charged residues and then a conserved hydrophobic residue (Fig. 1C; Fig. S1). The sequence conservation in the C-terminal tails contrasts with the limited sequence conservation in the C-terminal region of PilC/PilY proteins more generally, suggesting that these residues in the C-terminal tails have functional importance. Given the strong inter-species conservation of the C-terminal tail tryptophan residue and downstream hydrophobic residue, we performed site-directed mutagenesis to substitute PilC2 residues W1498 and I1501 with alanine residues (Δ*pilC1*-PilC2_1498A1501A_) to evaluate their effect on function (Fig. 5A). Mutation of these residues in PilC2 resulted in a slight reduction in extracellular pili levels compared to the parental control strain (Δ*pilC1*-PilC2_mark_) (Fig. 5B). Despite the modest effect on pili levels, strain Δ*pilC1*-PilC2_1498A1501A_ displayed a significant reduction in adherence to epithelial cells (Fig. 5C). Twitching motility levels in the Δ*pilC1*-PilC2_1498A1501A_ mutant were significantly lower than those in strain Δ*pilC1*-PilC2_mark_ and were comparable to the non-piliated (Δ*pilA1*) and PilC null (Δ*pilC1pilC2*) strains, suggesting that the PilC2 W1498 and I1501 residues are required for twitching motility (Fig. 5D). The transformation efficiency of strain Δ*pilC1*-PilC2_1498A1501A_ was significantly reduced compared to the parental strain (Fig. 5E). While strain Δ*pilC1*-PilC2_1498A1501A_ displayed increased transformation efficiency compared to strain Δ*pilC1pilC2*, this difference did not reach statistical significance. Taken together, these results indicate that dual mutation of PilC2 residues W1498 and I1501 to alanine residues significantly impacts T4P-mediated adherence, twitching motility, and natural transformation, while having only a modest effect on pilus biogenesis.

The AlphaFold Multimer model predicted that residues T193 and Q195 in the PilX globular domain β-sheet interact with conserved residues W1498 and I1501 in the PilC2 C-terminal tail (Fig. 5A). To test the functional significance of this predicted interaction, we performed site-directed mutagenesis to substitute PilX residues T193 and Q195 with alanine residues (Δ*pilX*-PilX_193A195A_) and assessed the resulting strain for T4P-dependent phenotypes. As shown in Fig. 5, strain Δ*pilX*-PilX_193A195A_ phenocopied strain Δ*pilC1*-PilC2_1498A1501A_ in terms of piliation (Fig. 5B), adherence (Fig. 5C), and twitching motility (Fig. 5D). We have previously demonstrated that deletion of *pilX* results in a complete loss of natural transformation (19). Interestingly, strain Δ*pilX*-PilX_193A195A_ was transformable and displayed a ~2 log reduction in transformation efficiency compared to the Δ*pilX*(*pilX*) parent strain. This result indicates that while significantly impaired, T4P containing mutant PilX_193A195A_ are able to promote natural transformation at a low level. Taken together, the observed phenocopying of strains Δ*pilC1*-PilC2_1498A1501A_ and Δ*pilX*-PilX_193A195A_ provides strong evidence for the predicted β-strand augmentation interaction between PilC2 and PilX.

## DISCUSSION

In this study, we identified a conserved C-terminal tail in the *K. kingae* PilC1 and PilC2 proteins that is required for multiple PilC-dependent functions, including surface piliation, adherence, twitching motility, and natural transformation. AlphaFold Multimer modeling predicts that these C-terminal tails insert into a hydrophobic pocket in the core minor pilin complex in *K. kingae* T4P. Extending this analysis to other bacteria with T4P revealed that this C-terminal tail is conserved and projects from the β-propeller domain into the core minor pilin complex. In each example, the C-terminal tail forms a β-strand that aligns with the β-sheet in the corresponding PilX/PilK homolog in the minor pilin complex, consistent with the structural arrangement called β-strand augmentation (25). The conserved C-terminus contains a tryptophan flanked by several hydrophobic residues. Point mutations of the *K. kingae* PilC2 C-terminal tryptophan (W1498) and isoleucine (I1501) residues completely abolished adherence and twitching motility and significantly reduced transformation efficiency. Mutations in the PilX amino acids predicted to interact with PilC2 W1498 and I1501 (PilX T193 and Q195) resulted in a phenotype that mimicked the PilC2 double mutant.

β-Strand augmentation refers to a protein-protein interaction that involves a β-strand from one protein interacting with the edge of a β-sheet in another, forming an extended β-sheet (29). The AlphaFold Multimer models suggested that PilC/PilY proteins contribute a β-strand that augments the β-sheet of the PilX/K homolog within the minor pilin complex. This structural arrangement is consistent with *in silico* models of the *M. xanthus* PilY1.3—minor pilin complex, in which a β-strand from PilY1.3 is predicted to pair with two β-strands of PilX3 (26). Analogous β-strand complementation mechanisms have been described in other pilus systems, including donor strand exchange during the assembly of chaperone-usher pili, with the N-terminal extension of an incoming pilus subunit interacting with the hydrophobic groove of the previously assembled subunit, displacing the chaperone donor strand (27, 30). When force is applied parallel to a β-strand augmentation interface, shearing of the interaction requires breaking of multiple hydrogen bonds at once (30). The alignment of the PilC/PilY C-terminal tail with a β-strand of the PilX/PilK homolog in our *in silico* models suggests that pilus retraction would apply force parallel to this interface, consistent with a mechanistically robust interaction that could retain PilC/PilY attachment to the minor pilin complex during T4P extension/retraction cycles.

Our data support a model in which the minor pilin complex serves as a structural scaffold where the conserved C-terminal tail of PilC/PilY inserts and functions as a mechanical adapter that attaches the adhesin to T4P. Loss of the C-terminal tail would therefore be expected to prevent stable loading of PilC/PilY onto T4P, causing defects in pilus extension and retraction. This framework explains why C-terminal truncation of *K. kingae* PilC1 and PilC2 prevented adherence, twitching motility, and natural transformation. Point mutations in the PilC2 C-terminal tail resulted in a partial inhibition of natural transformation, suggesting that several residues contribute cooperatively to mediate docking of PilC2 onto T4P. In *K. kingae*, the presence of two distinct PilC proteins may reflect a redundant docking mechanism, where the specific adhesin can be interchanged to engage different host ligands or surfaces in the environment. This work suggests testable models in which PilC1 and PilC2 either occupy the tip separately in different T4P machines or co-assemble on the same tip, and in which loading may be competitive or cooperative, allowing the organism to regulate which adhesins are presented to the environment in response to changing environmental signals. While deletion or mutation of the PilC1 or PilC2 C-terminal tail preventing proper loading onto the pilus is one explanation for the observed phenotypes, an alternate explanation is that this region of the protein is necessary for proper stabilization of the tip minor pilin complex. Further biochemical studies are necessary to distinguish between these possibilities.

During the drafting of this article, Ellison et al. (31) reported findings like ours in other T4P-expressing bacteria, including *N. gonorrhoeae* and *Acinetobacter baylyi*. These investigators demonstrated that the minor pilin PilK (PilX homolog) binds to the short C-terminal peptide of PilC/PilY1 proteins (what we call the C-terminal tails) through β-strand complementation (referred to as β-strand augmentation here) and that this dimer is recognized by the PilI-PilJ (PilV-PilW in the *K. kingae* nomenclature) minor pilin heterodimer to “license” T4P assembly. They also demonstrated that these β-strand augmentation/complementation interactions exist in more distantly related T4P systems and the evolutionarily related type II secretion system (T2SS). These observations highlight the importance of this protein-protein interaction for T4P function across diverse bacterial species. Our study complements the report by Ellison et al. by demonstrating the impact of C-terminal tail deletion or mutation on T4P-mediated functions, including adherence to host cells, twitching motility, and natural competence. In addition, our study further identifies key amino acid residues necessary for T4P biogenesis and function in both a representative PilC/PilY family member (*K. kingae* PilC2) and a PilX/PilK minor pilin family member (*K. kingae* PilX).

PilC/PilY family proteins are established virulence factors and have been proposed as attractive therapeutic targets in multiple T4P-expressing pathogens, given that they are surface-exposed and initiate infection. The identification of a conserved, mechanically critical docking interface between the PilC/PilY C-terminal tail and the minor pilin complex presents an opportunity for the development of anti-adhesion strategies based on small molecules or peptides that disrupt this interaction (32).

A limitation of this study is that the proposed β-strand augmentation interface is inferred from *in silico* modeling and genetic perturbation rather than direct structural and biophysical measurements. Future work will combine high-resolution cryo-electron microscopy, mutational scanning of the PilC-PilX β-strand interface, and single-molecule atomic force microscopy to define the structure, critical contact residues, and force-dependent binding properties of this interaction.

## MATERIALS AND METHODS

### Bacterial strains and culture conditions

*K. kingae* strains were grown on chocolate agar at 37°C supplemented with 5% CO_2_ for ~20 h. *Escherichia coli* strains were cultured on LB agar at 37°C or in LB broth shaking at 250 rpm at 37°C with 100 μg/mL ampicillin or 50 μg/mL kanamycin, as appropriate. All strains used in this study are listed in Table 1. All *K. kingae* mutants used in this study carry chromosomally encoded mutations. The general process for generating these mutant strains involves first creating a recombinant plasmid in *E. coli* that contains the deletion or mutation of interest that is marked with a nearby antibiotic resistance cassette, which is flanked by upstream and downstream *K. kingae* locus-specific targeting sequences. The construct is then linearized with restriction digestion and transformed into naturally competent *K. kingae*. The transformation reaction is plated on chocolate agar containing the appropriate antibiotic to select for transformants in which homologous recombination events occurred in both the upstream and downstream targeting regions, resulting in an allelic exchange event and introduction of the desired mutation. The transformants are screened by PCR and sequencing to confirm the mutant genotype. *K. kingae* strains were stored at −80°C in brain heart infusion (BHI) broth containing 20% glycerol, and *E. coli* strains were stored at −80°C in LB broth containing 15% glycerol.

**TABLE 1:** Bacterial strains and plasmids used in this work

Strain or plasmid	Description	Source
*K. kingae*		
KK03	Spreading/corroding derivative of clinical isolate 269-492	33
KK03Δ*pilA1*	KK03 with an *aphA3*-marked *pilA1* deletion	12
KK03Δ*pilC1pilC2*	KK03 with a *tetM*-marked *pilC1* deletion and an unmarked *pilC2* deletion	11
KK03Δ*pilC2*	KK03 with an unmarked *pilC2* deletion	11
KK03Δ*pilC1*	KK03 with a *tetM*-marked *pilC1* deletion	28
KK03Δ*pilC2*-PilC1_mark_	KK03 *pilC2* deletion strain with an *ermC* insertion immediately upstream of WT *pilC1*	11
KK03Δ*pilC2*-PilC1_Δ1350–1359_	KK03 Δ*pilC2*-PilC1_erm_ with a deletion of the 3′-region (a.a. 1350–1359) of *pilC1*	This study
KK03Δ*pilC1*-PilC2_mark_	KK03 *pilC1* deletion strain with an *aphA3* insertion immediately downstream of WT *pilC2*	11
KK03Δ*pilC1*-PilC2_Δ1493–1502_	KK03 Δ*pilC1*-PilC2_mark_ with a deletion of the 3′-region (a.a. 1493–1502) of *pilC2*	This study
KK03PilC2_1498A1501A_	KK03 with *aphA3*-marked mutagenesis of residues W1498A and I1501A	This study
KK03Δ*pilC1*-PilC2_1498A1501A_	KK03Δ*pilC1* with *aphA3*-marked mutagenesis of residues W1498A and I1501A	This study
KK03Δ*pilX*	KK03 with *ermC*-marked deletion of *pilX*	19
KK03Δ*pilX*(*pilX*)	KK03 with *ermC*-marked deletion of *pilX* complemented with the *pilX* under control of the *fimT* promoter	19
KK03Δ*pilX*-PilX_193A195A_	KK03Δ*pilX* complemented with mutant *pilX* encoding PilX containing point mutations T193A and Q195A	This study
*E. coli*		
Dh5α	F^−^ φ80d*lacZ*Δ*M15* Δ(*lacZYA-argF*)*U169 deoR recA1 endA1 hsdR17*(r_K_^−^ m_K_^+^) *phoA supE441 thi-1 gyrA96 relA1*	
Plasmids		
pUC19	High copy number cloning vector	34
pUC19/erm-PilC1	For introduction of *ermC-*marked PilC1 locus	28
pUC19/erm-PilC1_Δ1350–1359_	For introduction of deletion of 3′-region (a.a. 1350–1359) of *pilC1*	This study
pUC19/PilC2-kan	For introduction of *aphA3*-marked PilC2 locus	28
pUC19/PilC2_Δ1493–1502_-kan	For introduction of deletion of 3′-region (a.a. 1493–1502) of *pilC2*	This study
pUC19/PilC2_1498A1501A_	For introduction of mutations W1498A and I1501A in *pilC2*	This study
pComp-*pilX*	pComp-Kan complementation construct containing *pilX* for under control of the *fimT* promoter	19
pComp-PilX_193A195A_	pComp-*pilX* encoding mutant PilX_193A195A_	This study

### DNA cloning

For DNA cloning, DNA fragments were PCR amplified from the relevant target using Q5 HiFi Master Mix (New England Biolabs, Ipswich, MA, USA). Restriction enzymes and NEBuilder HiFi Assembly Master Mix were obtained from New England Biolabs. All plasmid constructs (listed in Table 1) were confirmed to be correct using a combination of restriction analysis, PCR, and sequencing using Sanger sequencing or long-read whole-plasmid sequencing by Plasmidsaurus (San Francisco, CA, USA).

### Site-directed mutagenesis

All primers used for the generation of mutants are listed in Table 2. To generate the PilC1_Δ1350–1359_ truncation, site-directed mutagenesis was performed using the QuikChange II XL kit (Agilent Technologies, Santa Clara, CA, USA) with primers PilC1Delta1350–1359F and PilC1Delta1350–1359R and the pUC19/erm-PilC1 plasmid as a template. To generate the PilC2_Δ1493–1502_ truncation, site-directed mutagenesis was performed using the QuikChange II XL kit with primers PilC2Delta1493–1502F and PilC2Delta1493–1502R and the pUC19/PilC2-kan plasmid as a template. To make two single point mutations (W1498A and I1501A) in PilC2, site-directed mutagenesis was performed using the QuikChange II XL kit with primers PilC2_1498A1501AF and PilC2_1498A1501AR and the pUC19/PilC2-kan plasmid as a template. To make two single point mutations (T193A and Q195A) in PilX, plasmid pComp-*pilX* was used as the template for site-directed mutagenesis with primers PilX_Q195A_F and PilX_Q195A_R, followed by mutagenesis with primers PilX_T193A_F and PilX_T193A_R. All mutagenesis PCR reactions were digested with DpnI for 1 h to remove template DNA and transformed into XL10-Gold Ultracompetent Cells (Agilent Technologies). Sequence-confirmed constructs were linearized with NdeI and transformed into *K. kingae* strains as previously described (35). As antibiotic resistance cassette markers are used for selection purposes when introducing mutations into PilC1 and PilC2, we used strains KK03Δ*pilC2*-PilC1_mark_ and KK03Δ*pilC1*-PilC2_mark_, respectively, as controls. The “mark” designation indicates that these strains contain the same chromosomal antibiotic resistance marker as the mutant strain but encode the wild-type PilC1 or PilC2 gene.

**TABLE 2:** Primers used in this work

Primer name	Sequence (5′→3′)
PilC1Delta1350–1359F	GTTGGTCGTCGTTGTGGTAAAAAAATACAATAATCAATTCATGCATTAGATAAAAAG
PilC1Delta1350–1359R	CTTTTTATCTAATGCATGAATTGATTATTGTATTTTTTTACCACAACGACGACCAAC
PilC2Delta1493–1502F	CACCAGCGACCCTGTGTAATCGTGATTAGCTG
PilC2Delta1493–1502R	CAGCTAATCACGATTACACAGGGTCGCTGGTG
PilC2_1498A1501AF	GACCCTGTGGTGAAACGTGTATCTGCGCGCGAGGCATTCTAATCGTGATTAGCTGAAAAA
PilC2_1498A1501AR	TTTTTCAGCTAATCACGATTAGAATGCCTCGCGCGCAGATACACGTTTCACCACAGGGTC
PilX_Q195A_F	AAAATAAAGATACAGTGGTTACGCTGGCAAGTTATGTTGAATTATTACGGTAAAG
PilX_Q195A_R	CTTTACCGTAATAATTCAACATAACTTGCCAGCGTAACCACTGTATCTTTATTTT
PilX_T193A_F	GCAAACCACTGTATCTTTATTTTGACC
PilX_T193A_R	TACAGTGGTTTGCCTGGCAAGTTATGTTGA

### Pilus preparations

*K. kingae* strains were incubated on chocolate agar at 37°C, 5% CO_2_ for 20 h. Bacterial growth was suspended in 12 mL of phosphate-buffered saline (PBS) to an OD_600_ of 0.8, vortexed at full speed for 1 min, and centrifuged at 4,000 × *g* for 30 min to pellet the bacteria. The bacterium-free supernatant was filtered through a 0.45 μm filter and then incubated with 20% ammonium sulfate on ice for 2 h to precipitate pili. The precipitated pili were collected by centrifugation at 20,000 × *g* for 20 min and were resuspended in SDS-PAGE loading buffer. Pilus preparations were separated on 15% SDS-PAGE gels, stained with Coomassie blue, and imaged with a Syngene XX6 G:Box system. Densitometry was performed using ImageJ on pilus preparations from three independent experiments. The band intensities of wild-type KK03 were normalized to 100%, and the quantification of the mutant pili levels is displayed as percent of KK03 ± standard error of the mean.

### Western blotting

Following SDS-PAGE separation and semi-dry transfer to nitrocellulose, the blots were blocked with 5% skim milk in PBS and probed with antiserum GP65 (17) (anti-PilA1; 1:20,000), antiserum Rab-128 (11) (anti-PilC1; 1:300), or antiserum GP25 (36) (anti-PilC2; 1:300). Horseradish peroxidase-conjugated anti-guinea pig or anti-rabbit secondary antibodies were used for detection, as appropriate. After exposure to a chemiluminescent substrate (SuperSignal West Pico Plus, Thermo Fisher Scientific, Waltham, MA, USA), the blots were imaged with a Syngene XX6 G:Box system.

### Eukaryotic cell lines

Chang cells (Wong-Kilbourne [D] of Chang conjunctiva, HeLa origin; ATCC CCL-20.2) were cultivated in modified Eagle’s medium (MEM) supplemented with 10% fetal bovine serum and 1% non-essential amino acids, as previously described (33).

### Bacterial adherence assays

Quantitative bacterial adherence assays were performed as described previously (12, 17, 28). Briefly, 1.8 × 10^5^ Chang cells were seeded into 24-well tissue culture-treated plates and incubated at 37°C with 5% CO_2_ for 18 h. Cell monolayers were fixed with 2% glutaraldehyde in 0.2 M sodium phosphate buffer (pH 7.4) for 90 min at 4°C. To remove the fixative, the wells were washed four times in Tris-buffered saline (pH 7.5). *K. kingae* strains were cultured on chocolate agar for ~20 h at 37°C with 5% CO_2_, the bacterial growth was resuspended in BHI media to an OD_600_ of 0.8, and 10 μL of the bacterial suspension was inoculated into 300 μL of pre-warmed 37°C MEM in the 24-well plates containing the fixed monolayers. The 24-well plate was centrifuged for 5 min at 1,000 rpm to facilitate bacterial contact with the monolayer and incubated at 37°C with 5% CO_2_ for 25 min. To remove non-adherent bacteria, the wells were washed 4× with PBS. Subsequently, 100 μL of trypsin-EDTA (0.05%) was added, and the plates were incubated for 20 min at 37°C with 5% CO_2_ to facilitate recovery of adherent bacteria. The recovered bacteria were plated on chocolate agar to determine CFU/mL. The percent adherence was calculated as a ratio of recovered bacteria to the inoculum.

### Twitching motility assays

Twitching motility assays were performed as previously described (28). *K. kingae* strains were cultured on chocolate agar for ~20 h at 37°C with 5% CO_2_, and the growth was resuspended in PBS to an OD_600_ of 0.8. One microliter of the bacterial suspension was stab inoculated into the bottom of tissue culture-treated petri dishes containing chocolate agar (1% agar). Plates were incubated for 72 h at 37°C with 5% CO_2_. Following incubation, the chocolate agar was removed, and the zone of bacterial spread was stained with 0.1% crystal violet. To reduce measurement bias, the area of crystal violet-stained bacterial spread was overlaid on an asterisk-like image in which six lines are arrayed like spokes emanating from a central point and separated by 60° angles. The stab inoculation point was centered on the image, and the diameter measurements from the edge of the bacterial spread zone along each of the three lines crossing the central stab inoculation point were recorded in millimeters.

### Natural transformation efficiency assays

Transformation efficiency assays were performed as previously described (11). *K. kingae* strains were cultured on chocolate agar for ~20 h at 37°C, 5% CO_2_, and the growth was resuspended in BHI media to an OD_600_ of 0.8. One microgram of plasmid DNA containing ~1,000 bp of upstream and downstream sequence flanking the *knh* gene and a kanamycin resistance cassette (*aphA3*), an erythromycin resistance cassette (*ermC*), or a tetracycline resistance cassette (*tetM*) in place of the *knh* gene was added to 250 μL of bacterial suspension in a 24-well plate. The 24-well plate was incubated at room temperature for 30 min, and then 250 μL of 20% lysed horse blood in BHI was added to each well. The mixture was incubated at 37°C, 5% CO_2_ for 2.5 h to allow for bacterial recovery before CFU plating on chocolate agar containing kanamycin (50 μg/mL), erythromycin (1 μg/mL), or tetracycline (2 μg/mL), as appropriate. The natural competence efficiency was calculated based on the ratio of recovered bacteria to inoculum.

### Bioinformatic analyses

Signal peptide predictions for AlphaFold prediction inputs were accomplished using SignalP 6.0 (https://services.healthtech.dtu.dk/services/SignalP-6.0/) (37). Genomic sequences and primary amino acid sequences were analyzed with nucleotide BLAST (BLASTn) and protein BLAST (BLASTp), respectively, hosted at the National Center for Biotechnology Information (https://www.ncbi.nlm.nih.gov/) (38). Sequence similarity/identity calculations and sequence alignments were performed using the EMBOSS Needle Pairwise Sequence Alignment tool (https://www.ebi.ac.uk/jdispatcher/psa/emboss_needle) (39). Structural prediction was accomplished using AlphaFold 3 and AlphaFold Multimer (https://www.alphafoldserver.com/) (40). AlphaFold 3 structural predictions were visualized using ChimeraX 1.9-rc2024.11.01 (41). Protein-protein interactions within AlphaFold-generated predicted models were inferred using the EMBL-EBI PDBePISA tool (https://www.ebi.ac.uk/pdbe/pisa/) (42). Sequence logo analysis was completed using WebLogo 3 (https://weblogo.threeplusone.com/) (43).

### Statistical analysis

Statistical analysis was conducted with GraphPad Prism version 11.0.0 using one-way analysis of variance with Bonferroni’s multiple comparisons test.

## SUPPLEMENTAL MATERIAL

Supplemental Material msphere.00464-26-s0001.pdf Supplemental table and figures.

ASM does not own the copyrights to Supplemental Material that may be linked to, or accessed through, an article. The authors have granted ASM a non-exclusive, world-wide license to publish the Supplemental Material files. Please contact the corresponding author directly for reuse.
