# Supplementary material for: Conserved C-terminal tails link PilC1 and PilC2 to the Kingella kingae type IV pilus through β-strand augmentation with the core minor pilin complex
Source: mSphere. 2026 Sep 21;11(10):e00464-26. doi: 10.1128/msphere.00464-26 (PMC13711793; doi:10.1128/msphere.00464-26)
Supplement: Supplementary file 1 [file msphere.00464-26-s0001.pdf]

Supplemental Material

Conserved C-terminal tails link PilC1 and PilC2 to the *Kingella kingae* type IV pilus through β-strand augmentation with the core minor pilin complex

Taylor A. Yount,[1,2] Eric A. Porsch,[2] Alexa Mihaita,[1] Aisling Macaraeg,[1] and Joseph W. St. Geme, III[1,2]

[1]Perelman School of Medicine, University of Pennsylvania, Philadelphia, PA
[2]Children's Hospital of Philadelphia, Philadelphia, PA

Table S1. AlphaFold 3 confidence metrics for all predicted structures

Figure	Modeled Structure(s)	Species (strain)	pTM	ipTM	Mean pLDDT	Interface PAE (Å)/Chain Pair	PilC/Y C-terminal tail pLDDT
Fig 1A, S2	PilC1 + FimTPilVWXE	*K. kingae* (KK03)	0.55	0.73	73.1	2.1 PilC1-PilX	81.0 (confident)
Fig 1B, 5A, S2	PilC2 + FimTPilVWXE	*K. kingae* (KK03)	0.53	0.73	72.0	1.9 PilC2-PilX	81.6 (confident)
S2 Fig	PilC1 + PilHIJKX	*N. meningitidis* (MC58)	0.72	0.76	79.9	1.8 PilC1-PilK	81.0 (confident)
S2 Fig	PilC2 + PilHIJKX	*N. meningitidis* (MC58)	0.76	0.76	80.8	1.8 PilC2-PilK	81.0 (confident)
S2 Fig	PilC1 + PilHIJKL	*N. gonorrhoeae* (FA1090)	0.73	0.77	79.2	1.6 PilC1-PilK	81.4 (confident)
S2 Fig	PilC2 + PilHIJKL	*N. gonorrhoeae* (FA1090)	0.73	0.77	79.1	1.7 PilC2-PilK	81.1 (confident)
S2 Fig	PilY1 + FimUPilVWXE	*P. aeruginosa* (PAO1)	0.74	0.78	80.7	1.4 PilY1-PilX	71.3 (confident)
S2 Fig	PilY1.3 + PilXWV + FimU	*M. xanthus* (DK1622)	0.79	0.87	80.5	1.2 PilY1.3-PilX	80.6 (confident)
S2 Fig	PilY1 + AVR58_03200 + PilV + AVR58_03190 + AVR58_03185 + AVR58_03175	*L. pneumophila* (ASM194158v1)	0.75	0.80	82.0	1.4 PilY1-AVR58_3190	79.7 (confident)
S3A Fig	PilC1 (full length)	*K. kingae* (KK03)	0.61	—	68.9	—	48.0 (likely disordered)
S3A Fig	PilC1Δ10 (tail removed)	*K. kingae* (KK03)	0.62	—	70.6	—	—
S3B Fig	PilC2 (full length)	*K. kingae* (KK03)	0.66	—	69.9	—	43.9 (likely disordered)
S3B Fig	PilC2Δ10 (tail removed)	*K. kingae* (KK03)	0.67	—	71.1	—	—

All structures were predicted with AlphaFold 3 (AlphaFold Server; one seed and five diffusion samples per job) and values are given for the top-ranked model. The pTM (predicted TM-score) and ipTM (interface predicted TM-score) values range from 0 to 1 with higher values indicating greater confidence in the overall fold and in the relative positioning of chains at interfaces, respectively (ipTM >0.8 indicates a high-confidence interface and 0.6–0.8 indicates a probable interface). The ipTM value applies only to multi-chain complexes. Mean pLDDT represents the mean per-atom predicted local distance difference test score across the model (>90 very high, 70–90 confident, 50–70 low, <50 very low/likely disordered). Interface PAE represents the minimum predicted aligned error (in Å) between the PilC/PilY protein and the minor pilin with the best-defined inter-subunit contact, where lower values indicate greater

confidence in the relative position of the two chains (<5 Å is considered confident). C-terminal tail pLDDT was calculated as the mean per-atom pLDDT of the last 10 residues of the PilC/PilY C-terminus. Dashes indicate metrics that do not apply to the corresponding structure.

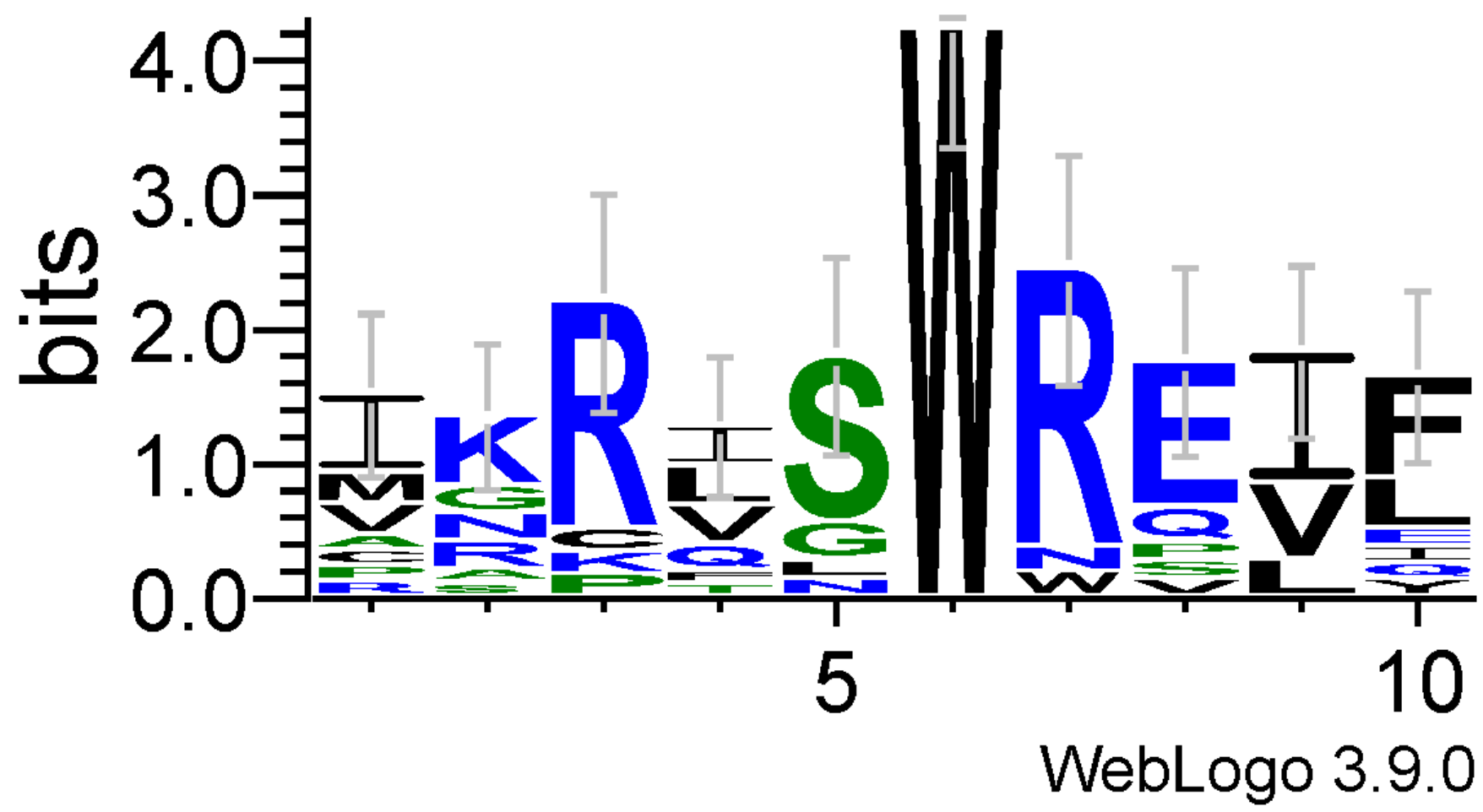

S1 Fig. Sequence logo of PilC/PilY family C-terminal tail conservation. Using the C-terminal 10 amino acids of *K. kingae* PilC1 and PilC2 as the aligned region and based on the multiple sequence alignment of PilC1/PilY family members displayed in Fig. 1C, a sequence logo conservation plot was generated using WebLogo3.

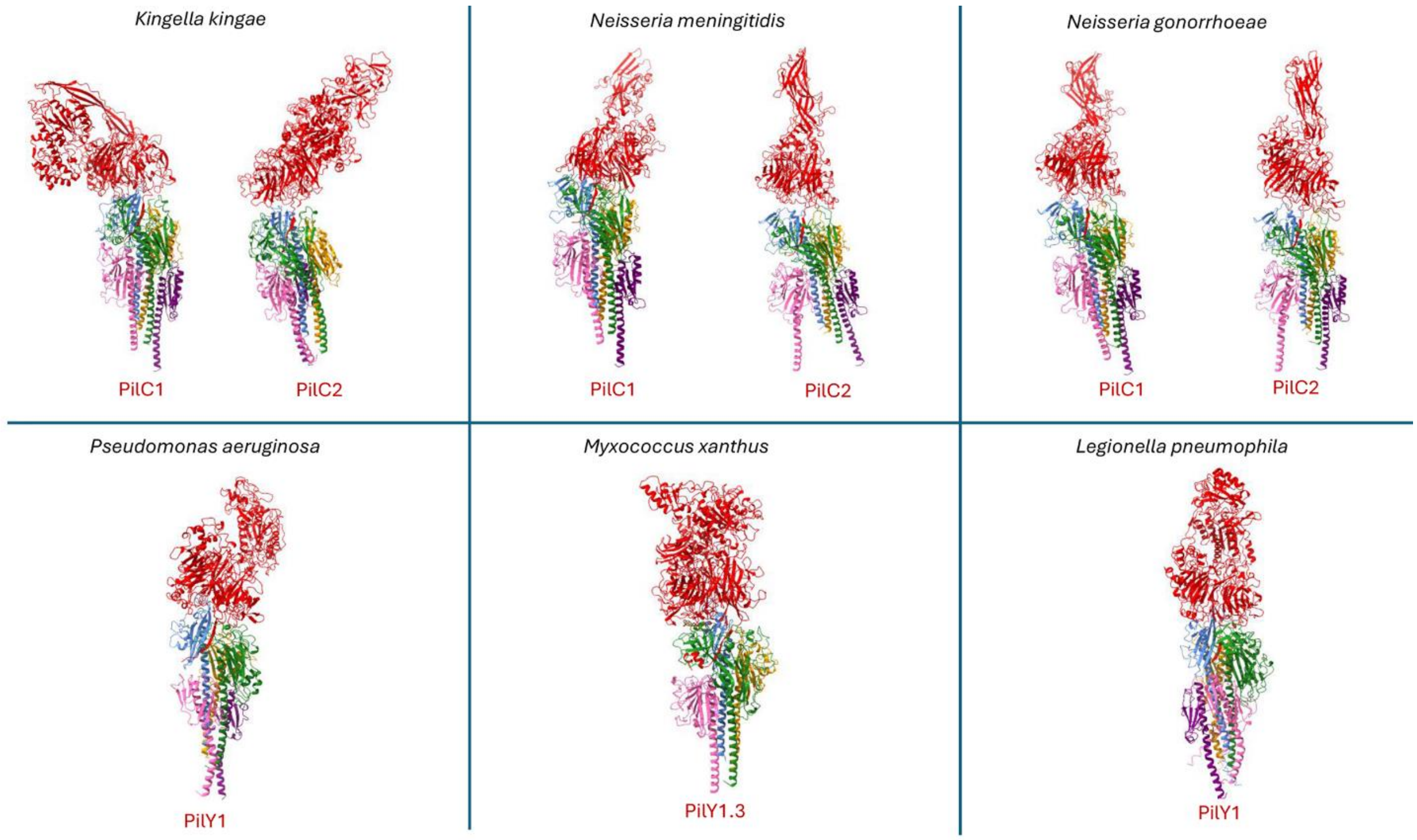

S2 Fig. AlphaFold Multimer predicted PilC/PilY-minor pilin complex interactions. AlphaFold Multimer was utilized to predict structures for PilC/PilY family members and their corresponding core minor pilins. The PilC/PilY family protein is labeled in red for each species and the minor pilins are color coded 5'-3' as they appear in their operons. For example, in *N. gonorrhoeae* PilHIJKX appear in pink, orange, green, blue, and purple, respectively. The reference strain for each species was used to access amino acid sequences for each protein: Top row: KK03, MC58, FA1090. Bottom row: PA01, DK1622, ASM194158v1. The AlphaFold confidence scores are listed in Table S1.

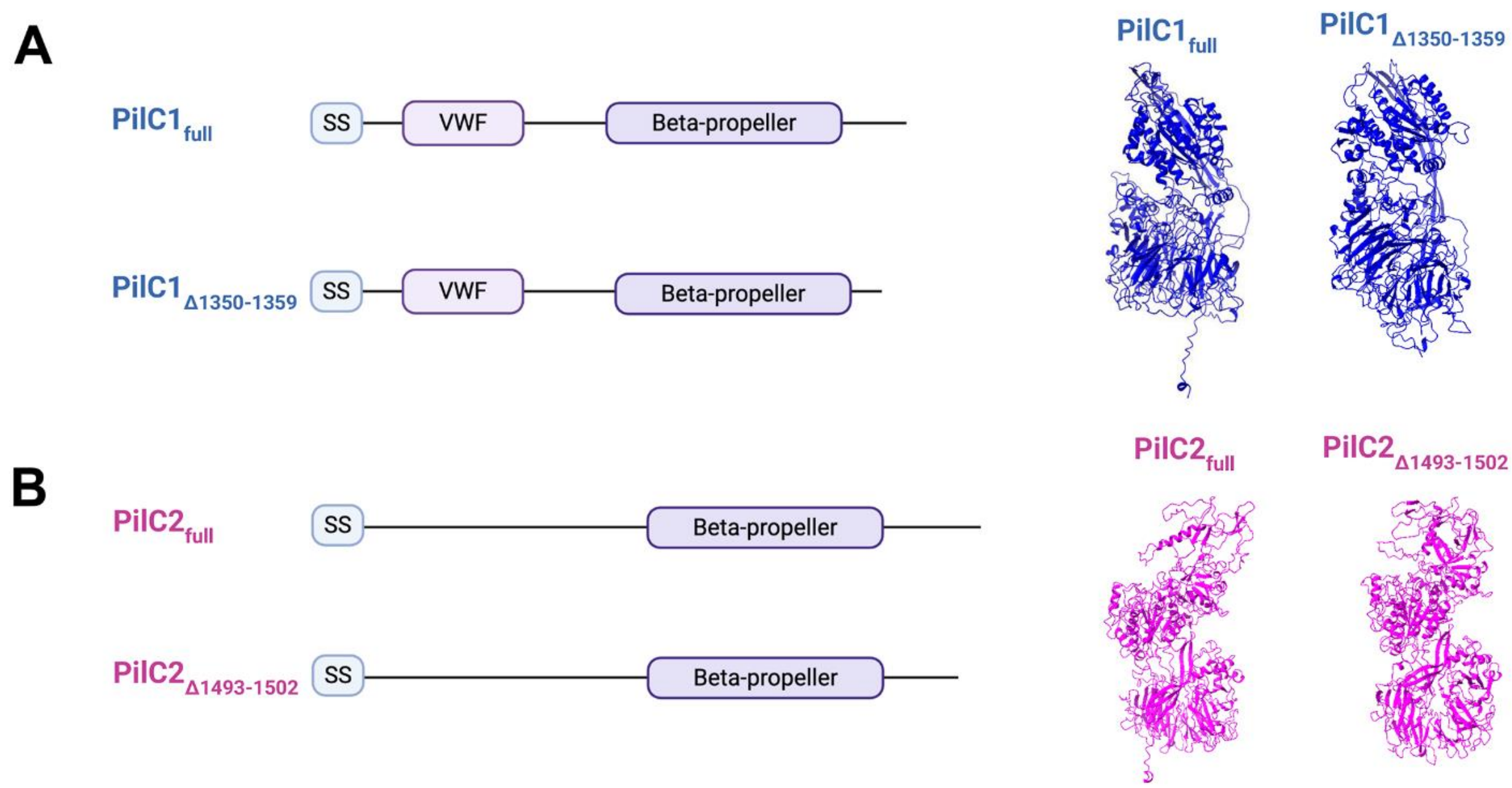

S3 Fig. PilC1 and PilC2 structural predictions. AlphaFold structural predictions for PilC1 (A) and PilC2 (B) in the presence and absence of the 10-residue C-terminal truncation. Protein schematics show the overall domain architecture of PilC1 and PilC2, including N-terminal Sec signal sequences (SS), N-terminal von Willebrand Factor Type A1 domains (VWF), and C-terminal β-propeller folds. The 10-residue truncation of both PilC1 and PilC2 is predicted to remove the tail projecting from the β-propeller domain without disrupting the β-propeller fold. The AlphaFold confidence scores are listed in Table S1.

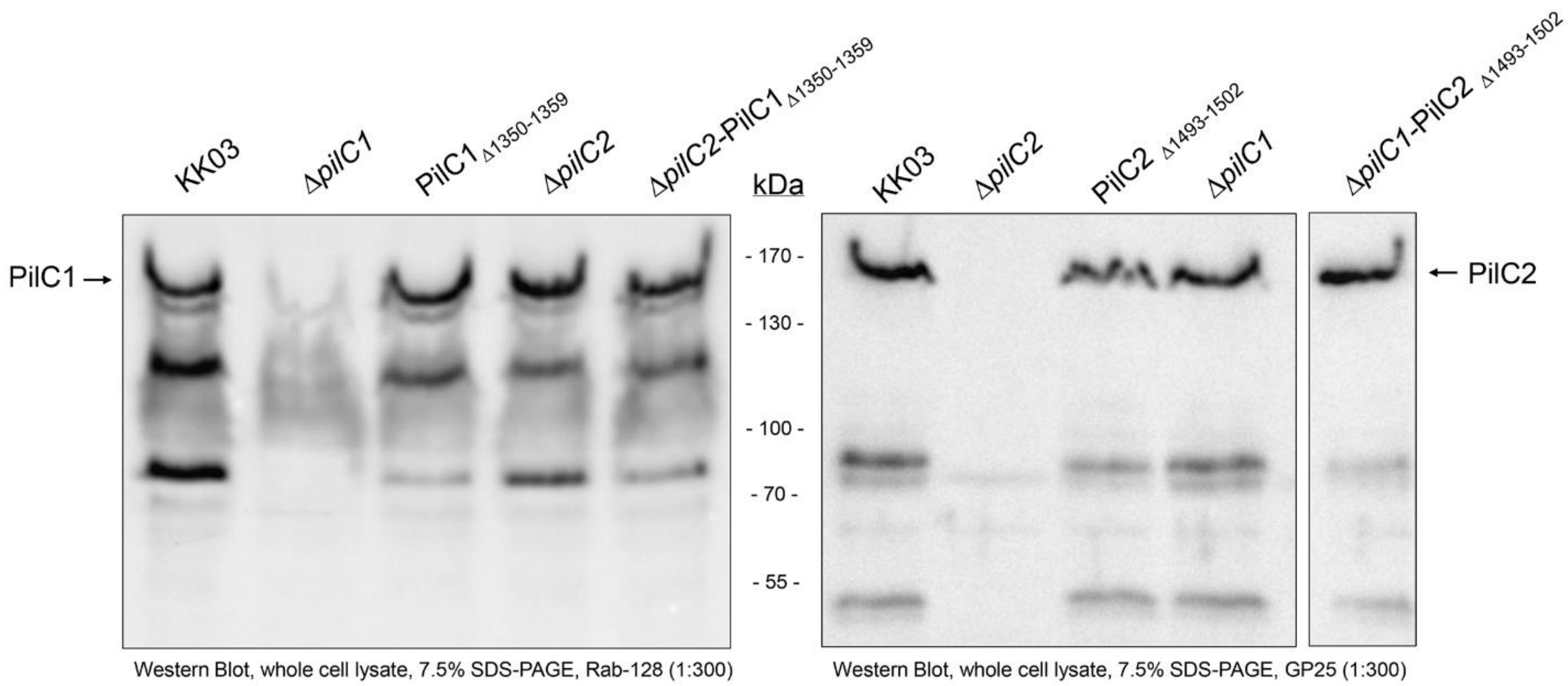

S4 Fig. Expression of PilC1$_{\Delta 1350\text{-}1359}$ and PilC2$_{\Delta 1493\text{-}1502}$. Western blotting analysis on cell lysates from PilC1 and PilC2 C-terminal truncation mutants. To assess for PilC1 and PilC2 expression, cell lysates were probed with PilC1 antiserum (Rab-128) or PilC2 antiserum (GP25), respectively. The molecular weight markers in kilodaltons (kDa) are indicated between the blot images.
